# Insights from quantitative and mathematical modelling on the proposed WHO 2030 goal for schistosomiasis

**DOI:** 10.12688/gatesopenres.13052.2

**Published:** 2019-11-19

**Authors:** NTD Modelling Consortium Schistosomiasis Group

**Keywords:** Schistosomiasis, WHO guidelines, Elimination as a public health problem, Mass drug administration, NTD Modelling Consortium

## Abstract

Schistosomiasis remains one of the neglected tropical diseases (NTDs) impacting millions of people around the world. The World Health Organization (WHO) recently proposed a goal of elimination as a public health problem (EPHP) for schistosomiasis to be reached by 2030. Current WHO treatment guidelines for achieving EPHP focus on targeting school-aged children. The NTD Modelling Consortium has developed mathematical models to study schistosomiasis transmission dynamics and the impact of control measures. Our modelling insights on *Schistosoma mansoni* have shown that EPHP is likely to be attainable in low to moderate prevalence settings using the current guidelines. However, as prevalence rises within high prevalence settings, EPHP is less likely to be achieved unless both school-aged children and adults are treated (with coverage levels increasing with the adult burden of infection). We highlight the challenges that are faced by treatment programmes, such as non-adherence to treatment and resurgence, which can hinder progress towards achieving and maintaining EPHP. Additionally, even though EPHP may be reached, prevalence can still be high due to persisting infections. Therefore, without interruption of transmission, treatment will likely have to continue to maintain EPHP. Further modelling work is being carried out, including extending our results to *S. haematobium*. By providing these modelling insights, we aim to inform discussions on the goals and treatment guidelines for schistosomiasis.

## Disclaimer

The views expressed in this article are those of the author(s). The opinions expressed herein are those of the authors and do not necessarily reflect the views of the World Health Organization. Publication in Gates Open Research does not imply endorsement by the Gates Foundation.

## Background

Schistosomiasis remains an endemic neglected tropical disease (NTD) affecting approximately 220 million people worldwide ^1^. It is an intestinal or urogenital disease caused predominantly by *Schistosoma mansoni* or *S. haematobium*. Individuals become infected when cercariae, released by freshwater snails, penetrate the skin during contact with contaminated water ^2^. The disease can result in anaemia, chronic pain, diarrhoea, and malnutrition, causing poor school performance and lower fitness ^3^. Donations of the treatment drug, praziquantel, are typically offered in school-based or community-wide mass drug administration (MDA) programmes for schistosomiasis.

The World Health Organization (WHO) has set goals of morbidity control and elimination as a public health problem (EPHP) for schistosomiasis to be reached by 2020 and 2025, respectively ^4, 5^ (defined in Table 1). There are recommended WHO treatment guidelines for achieving these goals based on the prevalence in school-aged children (SAC; aged 5–14 years old) prior to treatment. In low prevalence settings (≤10% SAC prevalence prior to treatment), MDA once every three years is recommended; in moderate prevalence settings (10–50% SAC prevalence prior to treatment), MDA once every two years is recommended; and in high prevalence settings (≥50% SAC prevalence prior to treatment), annual MDA is recommended ^4, 5^. MDA coverage has mainly focused on reaching 75% of SAC with treatment of adults at risk also recommended ^4, 5^. The WHO end goal for schistosomiasis is interruption of transmission (IOT) which is achieved once the incidence of infection is reduced to zero ^4, 5^. In May 2019, following a Global Schistosomiasis Alliance consultation meeting with its members and the WHO, there was support for the IOT goal with an interim and complementary goal of reducing the burden of schistosomiasis ^6^.

**Table 1.** Summary of modelling insights and challenges for reaching the WHO 2030 goal for *Schistosoma mansoni*.

Current WHO Goal (2020 Goal)	Morbidity control: <5% prevalence of heavy-intensity infections (eggs per gram ≥400) in school-aged children (SAC; 5–14 years old).
Proposed New WHO Goal (2030 Goal)	Elimination as a public health problem (EPHP): <1% prevalence of heavy-intensity infections in SAC. Note that this is the current 2025 goal.
Is the new goal technically feasible under the current disease strategy?	In low to moderate prevalence settings (<50% SAC prevalence prior to treatment), EPHP is likely to be achieved with 75% SAC-only treatment.
If not, what is required to achieve the goal?	As prevalence rises in high prevalence settings (≥50% SAC prevalence prior to treatment), EPHP becomes infeasible unless the disease strategy is scaled-up to treat both SAC and adults. Required coverage levels increase with the adult burden of infection.
Are current tools able to reliably measure the goal?	No; as Kato-Katz has low sensitivity at low prevalence levels, more sensitive diagnostics (able to measure prevalence and intensity of infection) will allow for smaller sample sizes and/or higher prevalence thresholds when measuring the goal.
What are the biggest unknowns?	Prevalence levels and intensity of infections across all age groups (i.e. full age profile of infection); levels of systematic non-adherence and ideal size of implementation unit; modelling insights on *S. haematobium* and other species.
What are the biggest risks?	Stopping treatment after achieving EPHP is highly likely to lead to resurgence of infection. Interruption of transmission (IOT) would alleviate the need for ongoing treatment. Potential risks posed by zoonotic reservoirs and drug resistance.

Mathematical models of transmission dynamics and the impact of control interventions have been developed to inform decision makers on the optimal treatment strategies which are required for achieving the WHO goals. The Gates-funded NTD Modelling Consortium brings together multiple institutional groups working on NTDs, including schistosomiasis. Modelling groups based at Imperial College London (ICL) and Case Western Reserve University (CWRU), along with other collaborators have led the recent work for schistosomiasis. A model comparison was carried out for the ICL and CWRU models, and a joint policy paper was also produced ^7, 8^. Due to knowledge gaps surrounding the epidemiology of schistosomiasis, the models have contrasting underlying assumptions leading to differences in model predictions ^8^. Despite these differences, the models generally agree on the treatment strategies required to achieve EPHP for *S. mansoni*, thereby strengthening the evidence for our model recommendations ^7^.

Moving towards the post-2020 goals, new WHO goals have been proposed for the NTDs to be reached by 2030. Currently, the proposed 2030 goal for schistosomiasis is EPHP. Using the insights that have been gained from recent modelling work on *S. mansoni*, we highlight the practical implications of EPHP (the timelines and feasibility of achieving EPHP) and the risks that need to be mitigated to maintain this goal. There is uncertainty around how reliable the current WHO definition of EPHP is for estimating a reduction in schistosomiasis-related morbidity as lower intensity infections may also be associated with significant morbidity ^3^. Further modelling will be required following revision of this goal by WHO as this may impact our recommended treatment strategies.

Note that the following sections focus on *S. mansoni* and Kato-Katz (as this is the currently recommended diagnostic technique ^9^). Additionally, the current WHO treatment guidelines and EPHP goal have been investigated here but these are currently under revision by WHO. Importantly, our modelling insights remain relevant as we highlight where the current guidelines are sufficient and where programmatic adaptations are needed for achieving the current EPHP goal (refer to Table 1 for a summary).

## Insights gained from quantitative and mathematical modelling analyses

**Figure 1.**
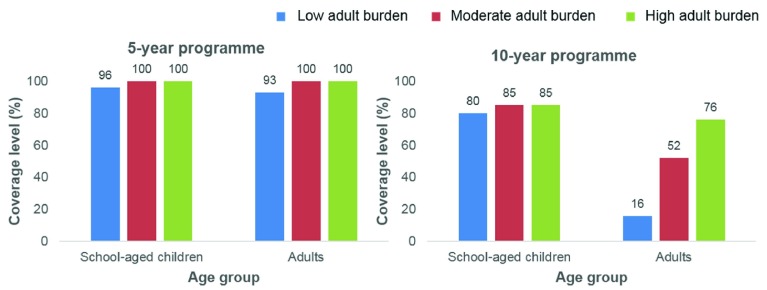
Coverage levels required to reach the WHO goal of elimination as a public health problem (EPHP) in a high prevalence setting (≥50% SAC baseline prevalence) within 5- and 10-year annual treatment programmes (assuming random coverage and no non-adherence). School-aged children (SAC) are 5–14 years old and adults are 15+ years old. This figure has been reproduced from 10 under a Creative Commons Attribution 4.0 International (CC BY 4.0) license.

Using models developed independently by ICL and CWRU, we investigated whether the currently recommended WHO guidelines (of 75% SAC-only treatment) are sufficient for achieving the EPHP goal for *S. mansoni*. Our modelling and data analyses showed that these guidelines are sufficient for reaching EPHP in low to moderate settings ^7, 10^. However, as prevalence rises within high settings, an increase and expansion in treatment coverage to include adults, as well as SAC, is required to reach EPHP with coverage levels dependent on the setting ^7, 10^ ( Table 2). As the burden of infection (intensity of transmission) in adults relative to SAC increases, the coverage levels needed to achieve EPHP increase ( Figure 1) ^10^. Coverage levels also increase if EPHP is to be achieved within a shorter amount of time ( Figure 1).

**Table 2.** Model recommended treatment strategies for achieving elimination as a public health problem (EPHP) in low to high prevalence settings. SAC refers to school-aged children aged 5–14 years old.

Prevalence in SAC prior to treatment	Model recommended treatment strategy for achieving EPHP
Low (<10%)	75% SAC treatment once every 3 years within 6 years ^7^.
Moderate (10%–50%)	75% SAC treatment once every 2 years for up to 5 years (this holds for low to high adult burdens of infection) ^10^.
High (≥50%)	As prevalence rises, SAC and adult annual treatment with coverage levels increasing with the adult burden of infection (coverage also increases as programme duration shortens; shown for 5–10 year programmes in Figure 1) ^10^.

Monitoring and evaluation (M&E) programmes are used to collect data to assess the progress of a treatment programme and to determine the appropriate treatment strategy. M&E data are typically collected from SAC as they are relatively easy to sample from. However, as the optimal treatment strategy for *S. mansoni* depends on the burden of infection in SAC and adults, M&E prevalence and infection intensity data need to be collected from a broader age-range ^10^. Our work has also shown that despite achieving EPHP, the prevalence may still be high due to light- to moderate-intensity infections persisting in SAC, in addition to all the infections remaining in pre-SAC and adults ^7, 10^. Therefore, stopping treatment after reaching EPHP poses a high risk of resurgence.

## Practical implications of the elimination as a public health problem goal

### Timelines and feasibility of achieving elimination as a public health problem

The treatment strategy required to achieve EPHP is determined by the epidemiological and ecological setting, such as the baseline prevalence/transmission intensity ^7, 10^. EPHP is technically feasible in all settings within 10 years provided that the appropriate treatment strategy is used. Table 2 shows the model recommended treatment strategies. Achieving and maintaining high coverage, adherence and treatment opportunities over each round of treatment is essential ^11^. Here, we have assumed treatment at random with full adherence at each round of MDA. Areas with poor school enrolment may benefit more from community-wide treatment ^12^.

### Measuring the elimination as a public health problem goal

To monitor and assess progress towards the EPHP goal, prevalence and infection intensity data are required from SAC (as the goal is defined by <1% prevalence of heavy-intensity infections in SAC). The goal is typically assessed by averaging the prevalence measured in five schools randomly sampled within a district ^13^. This approach does not take into account the high spatial heterogeneity and focality in *Schistosoma* prevalence. Taking implementation decisions at the district level using the currently proposed sampling strategy can lead to under- and over-treatment of SAC. Sampling fewer children in more schools has been shown to improve prevalence estimates, reducing under-treatment ^13^. Ongoing work on mapping protocols will allow for more precise targeted treatment.

Kato-Katz is currently the recommended diagnostic test, but there are relatively newer, more sensitive diagnostics available. Due to the reduced sensitivity of diagnostic techniques at low prevalence levels, the true prevalence is likely to be higher than the measured prevalence. Prevalence measured with Kato-Katz will be lower relative to that measured with more sensitive diagnostics, such as point-of-care circulating cathodic antigen (POC-CCA) tests, and this difference has been analysed, although the relationship between the two diagnostics remains unclear ^14– 16^. Therefore, the diagnostic technique used will impact the sampling strategy, with a more sensitive diagnostic likely facilitating the sampling of fewer people or the use of higher prevalence thresholds when measuring EPHP and furthermore IOT ^17^.

### Considerations of cost

Accurate, representative data on which age groups are infected are required to determine the most cost-effective treatment strategy, for example, only collecting data on high-risk adults can overestimate the benefit of community-wide treatment ^12^. The costs of diagnostic techniques also need to be considered. Although the traditional Kato-Katz diagnostic is seen as the cheaper test, given the increased sensitivity of POC-CCA, this may outweigh costs in the long term ^18^.

## Risks faced by treatment programmes

There are risks that need to be mitigated to achieve EPHP. Individuals with no access to treatment or those not taking treatment in any round of MDA (systematic non-adherers) may result in maintained transmission ^11, 19^. Due to systematic non-adherence, reported coverage may be higher than true coverage ^19^. Ideally data on adherence as well as coverage should be collected within M&E programmes as both will impact the outcome of treatment programmes ^19^.

M&E programmes focus on SAC, and may be biased to those who are treated, making it difficult to promptly identify a failing treatment programme. Therefore, it is vital that the M&E data collected is representative of each age group ^10, 12^. Manipulation of implementation unit size may mask persistent prevalence of challenging locations, such as hotspots. Guidance on mapping of schistosomiasis prevalence will aid in determining the optimal size of implementation units. Further risks which may reduce the effectiveness of treatment programmes are potential drug resistance (declining praziquantel efficacy following multiple rounds of treatment ^20^) and the presence of zoonotic reservoirs ^21, 22^. More insights are needed on such risks as more intensified treatment strategies than those currently recommended here may be required if they are present.

Following achievement of EPHP, infections may remain present in the population resulting in resurgence if treatment is stopped ^7, 10^. Pre-SAC can also be infected with schistosomes and a reservoir of infection may remain in this age group following MDA to other age groups. Development of a paediatric formulation of praziquantel for pre-SAC treatment would prevent this ^23^. Due to remaining infections, it is highly likely that treatment will still be needed to maintain control after achieving EPHP ^24^. Good water, sanitation and hygiene could aid in sustaining EPHP, allowing treatment to be scaled down ^25^.

## Moving towards interruption of transmission

To alleviate the need for ongoing treatment and to prevent resurgence, IOT is required after reaching EPHP ^2, 7, 10^. The transition of treatment programmes from EPHP to IOT will require reassessment of the treatment strategy, with consideration of complementary interventions such as behaviour change and snail control. Once very low prevalence levels have been achieved and a treatment programme is stopped, surveillance is needed to ensure that IOT has been achieved and that resurgence has not occurred. Currently, there is little guidance available for programmes when stopping treatment. Recently, the ICL model determined the post-treatment surveillance criteria for predicting IOT for *S. mansoni*. Results showed that a 1% Kato-Katz prevalence measured 2 years (or later) after stopping treatment across 200 individuals (randomly sampled from all age groups in a population of 500–1000 individuals), means IOT is 90% likely in the absence of re-introduction ^17^.

## Priority questions

**Table:** 

Priority issue / question identified in discussion with WHO	How can quantitative and mathematical modelling address this?
Re-run the models with the broad parameters of the new treatment guidelines.	New guidelines can be simulated in the model and followed through to determine if they are sufficient for achieving EPHP (as done previously for current guidelines ^7^).
Quantitative assessment of morbidity averted with continued treatment.	Modelling can simulate new guidelines to determine heavy-intensity infection prevalence and overall prevalence cases averted which can be related to morbidity averted.
How do we know when country settings can transition from EPHP to IOT? • What interventions are required? • What criteria are required? • Where possible? • What are the cost implications?	Modelling has been used to show the MDA treatment strategy required to achieve EPHP ^7, 10^. This can be extended to investigate the interventions required for transitioning to IOT. Modelling can then investigate the feasibility of sustaining EPHP versus moving to IOT. IOT prediction and post-MDA surveillance criteria have been determined for *S. mansoni* ^17^.

## Data availability

No data are associated with this article.
