# Supplementary material for: Insights from quantitative and mathematical modelling on the proposed WHO 2030 goal for schistosomiasis
Source: Gates Open Res. 2019 Nov 19;3:1517. Originally published 2019 Aug 16. [Version 2] doi: 10.12688/gatesopenres.13052.2 (PMC6820450; doi:10.12688/gatesopenres.13052.2)
Supplement: Peer review history

# Peer review: Reviewer response for version 2

**DOI:** 10.21956/gatesopenres.14237.r28258

**Published:** 2019-12-06

**Authors:** Stefanie Knopp

**Affiliations:** 1 Swiss Tropical and Public Health Institute, Basel, Switzerland

Thanks to the authors for the revisions made to their first version of this open letter. I remain with one comment that pertains to the content of current and future WHO guidelines, recommendations and/or goals, which I feel warrants consideration:

 In their letter, the authors refer to two WHO publications and indicate the therein suggested mass treatment recommendations for school-aged children as the basis for their modelling and to check whether elimination as public health problem can be achieved. This is fine. However, I feel it needs to be stated that these WHO recommended treatment strategies are for (morbidity) control of schistosomiasis, but that they are not the WHO recommended strategies to reach elimination as public health problem and interruption of transmission.

 I.e., the first document “Helminth control in school-aged children” published in 2011, focuses on schistosomiasis (morbidity) control only. There is no mention of “elimination” at all in this guide.

 The second document “Progress report 2001-2011 and strategic plan 2012-2020” published in 2013, distinguishes between treatment approaches for morbidity control (see Section 1.4 “Recommendations for morbidity control” and Table 1.1. in this section) and intervention strategies for elimination as public health problem and interruption of transmission (see Section 3.2 “Progression towards elimination of schistosomiasis” and Table 3.2. in this section). In their letter, the authors seem to refer to the approaches described in Table 1.1.. Again it needs to be clarified that this approach is the one recommended for (morbidity) control, but not for reaching elimination as public health problem.

 I have made an attempt to rephrase respective sentences in the revised PDF version of the letter so that they read correctly – please see the file here. The authors may consider to change accordingly. I have also inserted some more specific comments and track changes into the PDF version that the authors may want to address if they agree.

 Moreover, I would like the authors to carefully check in the published versions of the two WHO publications mentioned above plus the “proposed WHO goals 2021-2030”, whether at all and for which goal (morbidity control, elimination as public health problem, interruption of transmission) school-aged children are explicitly mentioned. As far as I can see, this is only the case in the recommendation for morbidity control, but not for elimination in any of the documents, and hence some sentences in the letter might need to be adapted accordingly.

I confirm that I have read this submission and believe that I have an appropriate level of expertise to confirm that it is of an acceptable scientific standard.

# Peer review: Reviewer response for version 1

**DOI:** 10.21956/gatesopenres.14179.r27696

**Published:** 2019-10-22

**Authors:** Darin Evans

**Affiliations:** 1 United States Agency for International Development (USAID), Washington, DC, USA

In this open letter, Toor and colleagues review the proposed WHO 2030 target for elimination of schistosomiasis as a public health problem (EPHP), defined as <1% heavy intensity (HI) infections, in the context of current WHO guidelines for preventive chemotherapy with praziquantel. Using mathematical models, they predict that, with 10 years of effective coverage of school-aged-children (i.e. 75%), communities with low or moderate prevalence schistosomiasis will be able to achieve the WHO defined threshold but that communities with higher prevalence and greater force of infection will require expanded treatment and greater coverage of adults. Similar findings were shown in a recent publication by Li *et al.* (2019 ^1^) though in their models expanded interventions, such as vector management, would be required. Importantly, in both cases, the authors recognize that once EPHP is achieved, transmission will continue, prevalence may remain high and treatment must continue. This instinctively draws into question the utility of setting <1% HI infection as a target if it will result in no programmatic change or action. In addition, the 1% HI threshold is an arbitrary one and evidence suggests that low to moderate intensity infections still have significant morbidity (King *et al.*, 2005 ^2^). The use of intensity of infection is a proxy indicator for morbidity and it relies on the inherently insensitive Kato-katz (KK).

 The authors discuss the availability of the more sensitive POC CCA test for *S. mansoni* in the context of comparing it to KK results but these should not be discounted as such tests are likely to become more common. Understanding better the use of such for identifying both transmission and morbidity will be important. Current studies such as the MORBID (Morbidity and Operational Research for Bilharziasis Implementation Decisions) project funded through COR-NTD will help inform better thresholds for programmatic decision making. Regardless of how EPHP is ultimately defined, this publication and others suggest that long-term efforts will be required to achieve and maintain this goal. This was recently highlighted by French *et al.* (2018 ^3^).

I confirm that I have read this submission and believe that I have an appropriate level of expertise to confirm that it is of an acceptable scientific standard.

## References

[1] : Improving public health control of schistosomiasis with a modified WHO strategy: a model-based comparison study. *The Lancet Global Health*.2019;7(10) : 10.1016/S2214-109X(19)30346-8 e1414-e1422 10.1016/S2214-109X(19)30346-8 PMC702498831537371

[2] : Reassessment of the cost of chronic helmintic infection: a meta-analysis of disability-related outcomes in endemic schistosomiasis. *The Lancet*.2005;365(9470) : 10.1016/S0140-6736(05)66457-4 1561-1569 10.1016/S0140-6736(05)66457-4 15866310

[3] : Schistosomiasis in Africa: Improving strategies for long-term and sustainable morbidity control. *PLoS Negl Trop Dis*.12(6) : 10.1371/journal.pntd.0006484 e0006484 10.1371/journal.pntd.0006484 29953454PMC6023105

# Response

**Published:** 2019-11-15

**Authors:** Jaspreet Toor

We thank Darin for his feedback and agree with the points raised. The following has been added in the background section: “There is uncertainty around how reliable the current WHO definition of EPHP is for estimating a reduction in schistosomiasis-related morbidity as lower intensity infections may also be associated with significant morbidity ^3^.”

 The following has been added to the risks faced by treatment programmes section: “Due to remaining infections, it is highly likely that treatment will still be needed to maintain control after achieving EPHP ^20a^.”

# Peer review: Reviewer response for version 1

**DOI:** 10.21956/gatesopenres.14179.r27700

**Published:** 2019-09-19

**Authors:** Stefanie Knopp

**Affiliations:** 1 Swiss Tropical and Public Health Institute, Basel, Switzerland

**Summary:**

- In this Open Letter article the authors reflect on results from quantitative and mathematical modelling that aimed to determine the impact of mass drug administration on the transmission and control of schistosomiasis and the impact of coverage in school-aged and adult populations to achieve elimination as public health problem (defined here as <1% heavy intensity infections in school-aged children) depending on the prevalence level. Challenges that schistosomiasis control programmes might face when they move towards or have achieved elimination as public health problem are presented. With the article, the authors aim to inform discussions on the WHO goals and treatment guidelines for schistosomiasis.
- The modelling results provide important insights into whether, when and how the prevalence of schistosomiasis can be reduced and thus can well contribute to inform WHO guidelines. However, as the article is currently presented, there seems to be confusion of what the currently published (until 17.9.2019) WHO guidelines recommend and for what level of control and elimination. The authors indicate mass drug administration in school-aged children as the intervention recommended by WHO to reach elimination as public health problem. To my understanding, WHO currently recommends to “adjust preventive chemotherapy and to use additional complementary public-health interventions” for reaching elimination as a public health problem (Reference 4 of the article). Also, the Global Schistosomiasis Alliance (GSA) provided invited feedback on the new proposed WHO goals for schistosomiasis post 2020. The goal proposed by WHO on April 10, 2019 was: "Elimination as a public health problem; Criteria to measure the achievement of the goal: Proportion of heavy intensity schistosomiasis infections <1%)". The feedback from GSA on May 2nd, 2019 was "… *the GSA strongly supports the proposal of the ambitious goal of interruption of transmission in selected countries and an interim and complementary goal of reducing the global burden of schistosomiasis disease…”. *It would be good if the authors can clarify on the guidelines and their actual content and also refer more to interruption of transmission as the ultimate goal.
- Please also consider my specific comments to the manuscript and peer review form:

 

**Is the rationale for the Open Letter provided in sufficient detail?**

- The rationale is provided. However, the authors might consider adding some more details. Please see my following comments:

Background:

1. *The authors write:* “The World Health Organization (WHO) has set goals of morbidity control and elimination as a public health problem (EPHP) for schistosomiasis to be reached by 2020 and 2025, respectively ^4^ (defined in Table 1).“ The authors might consider that the WHO also set a goal for interruption of transmission in selected countries, and present it to provide a complete picture of all goals set for 2020 and 2025.
2. *The authors write:* “There are recommended WHO treatment guidelines for achieving these goals based on the prevalence in school-aged children”. The authors might consider that in their strategic plan 2012-2020, the WHO recommends to “adjust preventive chemotherapy and to use additional complementary public-health interventions” for reaching elimination as a public health problem (World Health Organization, 2013 ^3^). Hence, in my view, the available guidelines do not focus on targeting school-aged children only. It seems to me that the authors refer to WHO recommendations for achieving morbidity control only, but not the actual guidelines for reaching elimination as public health problem. There are important differences in the approaches, which should be clarified.
3. *The authors write:* “Mathematical models of transmission dynamics and the impact of control interventions have been developed to inform decision makers on the optimal treatment strategies which are required for achieving the WHO goals”. The authors might clarify which goals exactly they write about.
4. *The authors write:* “Currently, the proposed 2030 goal for schistosomiasis is EPHP.” The authors might consider mentioning that WHO invited feedback and strategies for the proposed goals and that in the meantime in line with a consultation meeting, the Global Schistosomiasis Alliance (GSA) challenged that goal to some extent and responded to WHO as follows: “ *GSA strongly supports the proposal of the ambitious goal of interruption of transmission in selected countries and an interim and complementary goal of reducing the global burden of schistosomiasis disease.....” ( https://www.eliminateschisto.org/news-events/news/gsa-consultation-meeting-accelerating-progress-for-schistosomiasis-control-and).*
5. *The authors write: “*Note that the following sections focus on *S. mansoni* and Kato-Katz (as this is the currently recommended diagnostic technique).” The authors might provide a reference for this recommendation of Kato-Katz.
6. *The authors write: “*Additionally, the current WHO treatment guidelines have been investigated…”. The authors might consider highlighting which guidelines exactly they investigated and for what (i.e. morbidity control or elimination as public health problem) and how, and provide a reference to the guidelines.
7. *The authors write:* *“…*the currently recommended WHO guidelines (of 75% SAC-only treatment) are sufficient for achieving the EPHP goal for *S. mansoni*. *”* As indicated in my previous comments, the current guidelines indicate that to achieve elimination of schistosomiasis as a public health problem, WHO recommends to “adjust preventive chemotherapy and to use additional complementary public-health interventions” (World Health Organization, 2013 ^3^). Hence, it remains unclear, why the authors stick to recommendations for morbidity control and not elimination as a public health problem?
8. *The authors write: “*As the burden of infection in adults relative to SAC increases…”. The authors might define burden. Is it measured in intensity, worm numbers, DALYs or something else?

 

**Does the article adequately reference differing views and opinions?**

- My feeling is that some background on WHO guidelines for elimination as public health problem and interruption of transmission is missing or confused. Hence, it would be great if the authors can clarify that/why they went for modelling of morbidity control approaches rather than considering what is recommended by WHO to achieve elimination of public health problem as per the strategic plan 2012-2020.
- Moreover, there is a recent trial that underlines that in a mostly low prevalence setting, 5 years of biannual MDA are not sufficient to reach elimination as a public health problem in all sentinel sites and to achieve interruption of transmission (Knopp *et al.*, 2019a ^1^, Knopp *et al.*, 2019b ^2^). Additional operational research studies were published by members of the Schistosomiasis Consortium for Operational Research and Evaluation on controlling morbidity with different MDA approaches. The results and conclusions could be discussed in this letter in relation to the modelling results.

  

**Are all factual statements correct, and are statements and arguments made adequately supported by citations?**

- The authors might want to consider the following comments (the one pertaining to the Background was already outlined above):

Abstract:

1. *The authors write:* “The World Health Organization (WHO) recently proposed a goal of elimination as a public health problem (EPHP) for schistosomiasis to be reached by 2030. The authors might consider mentioning that WHO invited feedback and strategies for the proposed goals and that in the meantime in line with a consultation meeting, the Global Schistosomiasis Alliance (GSA) challenged that goal to some extent and responded to WHO as follows: “ *GSA strongly supports the proposal of the ambitious goal of interruption of transmission in selected countries and an interim and complementary goal of reducing the global burden of schistosomiasis disease.” ( https://www.eliminateschisto.org/news-events/news/gsa-consultation-meeting-accelerating-progress-for-schistosomiasis-control-and). *In my view, the advice of the GSA on the proposed WHO goals should also be highlighted somewhere in this article. “Recently” could be replaced by the date so that the reader knows when these new goals were proposed and whether they are still up to date or not.
2. *The authors write: “*Current WHO treatment guidelines for achieving EPHP focus on targeting school-aged children”. The authors might consider that in their strategic plan 2012-2020, WHO recommends to “adjust preventive chemotherapy and to use additional complementary public-health interventions” for reaching elimination as a public health problem” (Reference 4 of the article). Hence, the available guidelines do not focus on targeting school-aged children only?
3. *The authors write: *“The NTD Modelling Consortium has developed mathematical models to study schistosomiasis transmission dynamics and the impact of control measures”. The authors might consider highlighting which impact was measured (e.g. on prevalence or intensity) and to what control measures they refer (i.e. preventive chemotherapy or also measures such as snail control etc.).
4. *The authors write:* “Our modelling insights on *Schistosoma mansoni* have shown that EPHP is likely to be attainable in low to moderate prevalence settings using the current guidelines”. It would be nice if the authors can define how they define low and moderate (i.e. provide a prevalence range). Moreover, which guidelines do they refer to? As indicated in my 2. comment, the strategic plan 2012-2020 recommends to “adjust preventive chemotherapy and to use additional complementary public-health interventions”. Rather referring to the guidelines (which ones?), the authors might consider referring to their modelling approach on preventive chemotherapy for school-aged children.
5. *The authors write: *“However, as prevalence rises within higher settings…”. The authors might consider defining “higher setting”. The sentence needs revision.
6. *The authors write:* “…(with coverage levels increasing with the adult burden of infection).” It is not clear what the authors mean here. The authors might consider revising the part in brackets for more clarity.
7. *The authors write:* “…hinder progress towards achieving and maintaining EPHP”. The authors might consider that interruption of transmission has been suggested as goal, too (see my 1. Comment).
8. “EPHP” should be defined at the beginning so that the reader better understands the following sentence “Additionally, even though EPHP may be reached, prevalence can still be high”.
9. *The authors write:* “Therefore, without elimination of transmission, treatment will likely have to continue to maintain EPHP.” The authors might consider defining “treatment”. What treatment regimen do they have in mind? MDA?

 

 Background:

1. *The authors write:* “The World Health Organization (WHO) has set goals of morbidity control and elimination as a public health problem (EPHP) for schistosomiasis to be reached by 2020 and 2025, respectively ^4^ (defined in Table 1).” The authors might consider that the WHO also set goals for interruption of transmission in selected countries, to provide a complete picture of the goals set for 2020 and 2025.
2. *The authors write:* “There are recommended WHO treatment guidelines for achieving these goals based on the prevalence in school-aged children”. The authors might consider that in their strategic plan 2012-2020, the WHO recommends to “adjust preventive chemotherapy and to use additional complementary public-health interventions” for reaching elimination as a public health problem (World Health Organization, 2013 ^3^). Hence, in my view, the available guidelines do not focus on targeting school-aged children only. It seems to me that the authors refer to WHO recommendations for achieving morbidity control only, but not the actual guidelines for reaching elimination as public health problem. There are important differences in the approaches, which should be clarified.
3. *The authors write:* “Mathematical models of transmission dynamics and the impact of control interventions have been developed to inform decision makers on the optimal treatment strategies which are required for achieving the WHO goals”. The authors might clarify which goals exactly they write about.
4. *The authors write: *“Currently, the proposed 2030 goal for schistosomiasis is EPHP.” The authors might consider mentioning that WHO invited feedback and strategies for the proposed goals and that in the meantime in line with a consultation meeting, the Global Schistosomiasis Alliance (GSA) challenged that goal to some extent and responded to WHO as follows: “ *GSA strongly supports the proposal of the ambitious goal of interruption of transmission in selected countries and an interim and complementary goal of reducing the global burden of schistosomiasis disease.” ( https://www.eliminateschisto.org/news-events/news/gsa-consultation-meeting-accelerating-progress-for-schistosomiasis-control-and).*
5. *The authors write: “*Note that the following sections focus on *S. mansoni* and Kato-Katz” (as this is the currently recommended diagnostic technique). The authors might provide a reference for this recommendation of Kato-Katz.
6. *The authors write: “*Additionally, the current WHO treatment guidelines have been investigated...”. The authors might consider highlighting which guidelines exactly they investigated and for what (i.e. morbidity control or elimination as public health problem) and how, and provide a reference to the guidelines.
7. *The authors write: “…*the currently recommended WHO guidelines (of 75% SAC-only treatment) are sufficient for achieving the EPHP goal for *S. mansoni*. *” As indicated in my previous comments, the current guidelines indicate that to achieve elimination of schistosomiasis as a public health problem, *WHO recommends to “adjust preventive chemotherapy and to use additional complementary public-health interventions” (World Health Organization, 2013 ^3^). Hence, it remains unclear, why the authors stick to recommendations for morbidity control and not elimination as a public health problem?
8. *The authors write: “*As the burden of infection in adults relative to SAC increases…”. The authors might define burden. Is it measured in intensity, worm numbers, DALYs or something else?

 

 Practical implications:

  

1. *The authors write:* “EPHP is technically feasible in all settings within 10 years provided that the appropriate treatment strategy is used...”. A recent study on *S. haematobium* in Zanzibar, a setting targeted for elimination as public health problem and interruption of transmission, showed that despite 5 years of bi-annual MDA in schoolchildren with high coverage and adults with moderate compliance, elimination as public health problem was not reached in all sentinel sites, probably due to heterogeneity in transmission and some remaining pockets with high transmission potential, where people got reinfected rapidly (Knopp *et al.*, 2019a ^1^, Knopp *et al.*, 2019b ^2^). The authors might consider validating their model based on available field data.
2. *The authors write:* “To monitor and assess progress towards the EPHP goal, prevalence and infection intensity data are required from SAC (as the goal is defined by <1% prevalence of heavy-intensity infections in SAC).” The authors might consider adding a reference. When checking the proposed goals published by WHO at https://www.who.int/neglected_diseases/news/NTD-Roadmap-targets-2021-2030.pdf?ua=1, I did not see any mention of “SAC“. The current guidelines define elimination as public health problem as “<1% heavy infection intensities in all sentinel sites” (World Health Organization, 2013 ^3^).
3. *The authors write:* “The goal is currently assessed by averaging the prevalence measured in five schools randomly sampled within a district.” Is measuring prevalence in 5 schools related to the modelling approach of the authors or is it a recommendation by WHO to? If the latter, the authors might consider providing a reference.
4. *The authors write:* “Pre-SAC can also be infected with schistosomiasis…”. The authors might consider that a patient is not infected with a disease but with the disease-causing agent, in this case schistosomes.
5. *The authors write:* “Development of a paediatric formulation of praziquantel for pre-SAC treatment would prevent this”. The authors might consider that not only the development but finally only the large-scale application of the paediatric praziquantel would reduce a potential reservoir in PSAC.
6. *The authors write:* “Due to remaining infections, it is highly likely that treatment will still be needed after achieving EPHP”. Again, it would be nice if the authors can clarify what is meant with “treatment”.
7. *The authors write:* “To alleviate the need for ongoing treatment and to prevent resurgence, EOT is required after reaching EPHP.” The authors might consider indicating that this is/was one of the goals of WHO for selected countries until 2025 and that also “ *GSA strongly supports the proposal of the ambitious goal of interruption of transmission in selected countries”.*
8. *The authors write:* “The transition of treatment programmes from EPHP to EOT will require reassessment of the treatment strategy.” The authors might consider clarifying if they indeed refer to treatment strategies only, or broader intervention strategies, i.e. including also snail control, WASH, behaviour change, surveillance-response, etc.

 

 Table 1:

1. *The authors write:* “Elimination as a public health problem (EPHP): <1% prevalence of heavy-intensity infections in SAC”. Note that this is the current 2025 goal. To my knowledge, the current goal is not referring to SAC but sentinel sites?
2. *The authors write:* “In low to moderate prevalence settings (<50% SAC prevalence prior to treatment), EPHP is likely to be achieved with 75% SAC-only treatment.” For how many years is SAC needed to be treated?

 

 Priority questions:

- Validation of the models with real data from different prevalence and intensity settings could be one additional aim.

 

**Is the Open Letter written in accessible language?**

- Some sentences need to be carefully revised, but generally fine.

 

**Where applicable, are recommendations and next steps explained clearly for others to follow?**

- Yes.

I confirm that I have read this submission and believe that I have an appropriate level of expertise to confirm that it is of an acceptable scientific standard, however I have significant reservations, as outlined above.

## References

[1] : A 5-Year intervention study on elimination of urogenital schistosomiasis in Zanzibar: Parasitological results of annual cross-sectional surveys. *PLoS Negl Trop Dis*.2019;13(5) : 10.1371/journal.pntd.0007268 e0007268 10.1371/journal.pntd.0007268 31059495PMC6502312

[2] : Evaluation of integrated interventions layered on mass drug administration for urogenital schistosomiasis elimination: a cluster-randomised trial. *The Lancet Global Health*.2019;7(8) : 10.1016/S2214-109X(19)30189-5 e1118-e1129 10.1016/S2214-109X(19)30189-5 PMC662442431255591

[3] : Schistosomiasis: progress report 2001-2011, strategic plan 2012-2020. *World Health Organization*.2013; Reference source

# Response

**Published:** 2019-11-15

**Authors:** Jaspreet Toor

*We thank Stefanie for her comments and have responded to them in more detail below.*

**Summary:**

In this Open Letter article the authors reflect on results from quantitative and mathematical modelling that aimed to determine the impact of mass drug administration on the transmission and control of schistosomiasis and the impact of coverage in school-aged and adult populations to achieve elimination as public health problem (defined here as <1% heavy intensity infections in school-aged children) depending on the prevalence level. Challenges that schistosomiasis control programmes might face when they move towards or have achieved elimination as public health problem are presented. With the article, the authors aim to inform discussions on the WHO goals and treatment guidelines for schistosomiasis.

The modelling results provide important insights into whether, when and how the prevalence of schistosomiasis can be reduced and thus can well contribute to inform WHO guidelines. However, as the article is currently presented, there seems to be confusion of what the currently published (until 17.9.2019) WHO guidelines recommend and for what level of control and elimination. The authors indicate mass drug administration in school-aged children as the intervention recommended by WHO to reach elimination as public health problem. To my understanding, WHO currently recommends to “adjust preventive chemotherapy and to use additional complementary public-health interventions” for reaching elimination as a public health problem (Reference 4 of the article). Also, the Global Schistosomiasis Alliance (GSA) provided invited feedback on the new proposed WHO goals for schistosomiasis post 2020. The goal proposed by WHO on April 10, 2019 was: "Elimination as a public health problem; Criteria to measure the achievement of the goal: Proportion of heavy intensity schistosomiasis infections <1%)". The feedback from GSA on May 2nd, 2019 was "…the GSA strongly supports the proposal of the ambitious goal of interruption of transmission in selected countries and an interim and complementary goal of reducing the global burden of schistosomiasis disease…”. It would be good if the authors can clarify on the guidelines and their actual content and also refer more to interruption of transmission as the ultimate goal.

Please also consider my specific comments to the manuscript and peer review form.

In the background section, the end goal of interruption of transmission has been stated more clearly (had previously been referred to as elimination of transmission throughout the letter). The last paragraph of the background section has been edited to make clearer that we have investigated current WHO guidelines in relation to the current EPHP goal. We have also noted in this paragraph that the current WHO goals and guidelines are under revision. Also see response * to comment below.

**Is the rationale for the Open Letter provided in sufficient detail?**

The rationale is provided. However, the authors might consider adding some more details. Please see my following comments.

**Does the article adequately reference differing views and opinions?**

-My feeling is that some background on WHO guidelines for elimination as public health problem and interruption of transmission is missing or confused. Hence, it would be great if the authors can clarify that/why they went for modelling of morbidity control approaches rather than considering what is recommended by WHO to achieve elimination of public health problem as per the strategic plan 2012-2020.

* As stated in the background section for morbidity control and EPHP, the WHO currently recommends 75% SAC treatment with treatment of adults at risk also being recommended. However, reported coverage shows that typically SAC are treated (ref 1); country programme managers have also said that they typically only have praziquantel available for SAC and not adults. Hence, we have followed treating 75% of SAC at the recommended MDA frequency (as detailed in background section; Table 1.1 recommended treatment strategy for schistosomiasis in WHO reference 4 of paper which is for morbidity control and EPHP). WHO reference 4a has also been added which states that the WHO target is to reach at least 75% SAC.

-Moreover, there is a recent trial that underlines that in a mostly low prevalence setting, 5 years of biannual MDA are not sufficient to reach elimination as a public health problem in all sentinel sites and to achieve interruption of transmission (Knopp et al., 2019a ^1^, Knopp et al., 2019b ^2^). Additional operational research studies were published by members of the Schistosomiasis Consortium for Operational Research and Evaluation on controlling morbidity with different MDA approaches. The results and conclusions could be discussed in this letter in relation to the modelling results.

** We are presenting these results as modelling insights and are aware of the risks faced by treatment programmes which will mean that our model recommendations are too optimistic if such risks are faced.

*We have clarified the model assumptions on coverage and adherence in the timelines section: “Here, we have assumed treatment at random with full adherence at each round of MDA”. We have added “More insights are needed on such risks as more intensified treatment strategies than those currently recommended here may be required if they are present” to the risks faced by treatment programmes section (also within our biggest unknowns and risks in Table 1).*

**Are all factual statements correct, and are statements and arguments made adequately supported by citations?**

-The authors might want to consider the following comments:

**Abstract:**

1. The authors write: “The World Health Organization (WHO) recently proposed a goal of elimination as a public health problem (EPHP) for schistosomiasis to be reached by 2030. The authors might consider mentioning that WHO invited feedback and strategies for the proposed goals and that in the meantime in line with a consultation meeting, the Global Schistosomiasis Alliance (GSA) challenged that goal to some extent and responded to WHO as follows: “GSA strongly supports the proposal of the ambitious goal of interruption of transmission in selected countries and an interim and complementary goal of reducing the global burden of schistosomiasis disease.” (https://www.eliminateschisto.org/news-events/news/gsa-consultation-meeting-accelerating-progress-for-schistosomiasis-control-and). In my view, the advice of the GSA on the proposed WHO goals should also be highlighted somewhere in this article. “Recently” could be replaced by the date so that the reader knows when these new goals were proposed and whether they are still up to date or not.

*^ The following has been added to the background: “In May 2019, following a Global Schistosomiasis Alliance consultation meeting with its members and the WHO, there was support for the IOT goal with an interim and complementary goal of reducing the burden of schistosomiasis ^4b^.” Many of the abstract comments have been addressed throughout the text due to the word limit for the abstract.*

2. The authors write: “Current WHO treatment guidelines for achieving EPHP focus on targeting school-aged children”. The authors might consider that in their strategic plan 2012-2020, WHO recommends to “adjust preventive chemotherapy and to use additional complementary public-health interventions” for reaching elimination as a public health problem” (Reference 4 of the article). Hence, the available guidelines do not focus on targeting school-aged children only?

This is explained in the background section: “MDA coverage has mainly focused on reaching 75% of SAC with treatment of adults at risk also recommended.” We are using the WHO recommended treatment strategy shown in Table 1.1 and 2.2 in reference 4 and 4a of paper, respectively. This refers to treatment of SAC and adults at risk. To maintain clarity of which WHO guidelines we have analysed we are keeping the focus on targeting of SAC. Also see response * to comment above.

3. The authors write: “The NTD Modelling Consortium has developed mathematical models to study schistosomiasis transmission dynamics and the impact of control measures”. The authors might consider highlighting which impact was measured (e.g. on prevalence or intensity) and to what control measures they refer (i.e. preventive chemotherapy or also measures such as snail control etc.).

*Control measures investigated vary within the consortium. ICL looks at MDA, whereas, CWRU looks at MDA and snail control. We look at both prevalence and intensity. We feel this is too detailed to add to the abstract and have made it clear throughout the letter that we are focussing on MDA-only here. Further model details are available in the cited references, e.g. reference 6 in letter.*

4. The authors write: “Our modelling insights on Schistosoma mansoni have shown that EPHP is likely to be attainable in low to moderate prevalence settings using the current guidelines”. It would be nice if the authors can define how they define low and moderate (i.e. provide a prevalence range). Moreover, which guidelines do they refer to? As indicated in my 2. comment, the strategic plan 2012-2020 recommends to “adjust preventive chemotherapy and to use additional complementary public-health interventions”. Rather referring to the guidelines (which ones?), the authors might consider referring to their modelling approach on preventive chemotherapy for school-aged children.

*Using current WHO guidelines as stated. We use WHO definitions for low, moderate and high prevalence settings which are explained in the background section and tables 1-2.*

5. The authors write: “However, as prevalence rises within higher settings…”. The authors might consider defining “higher setting”. The sentence needs revision.

*Edited to “as prevalence rises within high prevalence settings…” Using WHO definition for high prevalence settings which is defined in background section and tables 1-2.*

6. The authors write: “…(with coverage levels increasing with the adult burden of infection).” It is not clear what the authors mean here. The authors might consider revising the part in brackets for more clarity.

*This is explained in the insights gained from quantitative and mathematical modelling analyses section and shown in figure 1.*

7. The authors write: “…hinder progress towards achieving and maintaining EPHP”. The authors might consider that interruption of transmission has been suggested as goal, too (see my 1. Comment).

*IOT has been mentioned in the background: “The WHO end goal for schistosomiasis is interruption of transmission (IOT) which is achieved once the incidence of infection is reduced to zero ^4, 4a^.”*

8. “EPHP” should be defined at the beginning so that the reader better understands the following sentence “Additionally, even though EPHP may be reached, prevalence can still be high”.

*EPHP has been defined in Table 1 at its first mention in the letter.*

9. The authors write: “Therefore, without elimination of transmission, treatment will likely have to continue to maintain EPHP.” The authors might consider defining “treatment”. What treatment regimen do they have in mind? MDA?

*Treatment has been left broad here as it does not mean only MDA necessarily, e.g. could include WASH as mentioned in the risks faced by treatment programmes section.*

**Background:**

1. The authors write: “The World Health Organization (WHO) has set goals of morbidity control and elimination as a public health problem (EPHP) for schistosomiasis to be reached by 2020 and 2025, respectively ^4^ (defined in Table 1).” The authors might consider that the WHO also set goals for interruption of transmission in selected countries, to provide a complete picture of the goals set for 2020 and 2025.

*This has been mentioned in the background: “The WHO end goal for schistosomiasis is interruption of transmission (IOT) which is achieved once the incidence of infection is reduced to zero ^4, 4a^.”*

2. The authors write: “There are recommended WHO treatment guidelines for achieving these goals based on the prevalence in school-aged children”. The authors might consider that in their strategic plan 2012-2020, the WHO recommends to “adjust preventive chemotherapy and to use additional complementary public-health interventions” for reaching elimination as a public health problem (World Health Organization, 2013 ^3^). Hence, in my view, the available guidelines do not focus on targeting school-aged children only. It seems to me that the authors refer to WHO recommendations for achieving morbidity control only, but not the actual guidelines for reaching elimination as public health problem. There are important differences in the approaches, which should be clarified.

See responses to abstract comment 2 and comment *.

3. The authors write: “Mathematical models of transmission dynamics and the impact of control interventions have been developed to inform decision makers on the optimal treatment strategies which are required for achieving the WHO goals”. The authors might clarify which goals exactly they write about.

*The last paragraph of the background section has been edited to make clearer that we have investigated current WHO guidelines in relation to the current EPHP goal.*

4. The authors write: “Currently, the proposed 2030 goal for schistosomiasis is EPHP.” The authors might consider mentioning that WHO invited feedback and strategies for the proposed goals and that in the meantime in line with a consultation meeting, the Global Schistosomiasis Alliance (GSA) challenged that goal to some extent and responded to WHO as follows: “GSA strongly supports the proposal of the ambitious goal of interruption of transmission in selected countries and an interim and complementary goal of reducing the global burden of schistosomiasis disease.” ( https://www.eliminateschisto.org/news-events/news/gsa-consultation-meeting-accelerating-progress-for-schistosomiasis-control-and).

*See response ^.*

5. The authors write: “Note that the following sections focus on S. mansoni and Kato-Katz” (as this is the currently recommended diagnostic technique). The authors might provide a reference for this recommendation of Kato-Katz.

*Reference 6a has been added for this.*

6. The authors write: “Additionally, the current WHO treatment guidelines have been investigated...”. The authors might consider highlighting which guidelines exactly they investigated and for what (i.e. morbidity control or elimination as public health problem) and how, and provide a reference to the guidelines.

See response to comment 3 above, comment 2 in abstract and *.

7. The authors write: “…the currently recommended WHO guidelines (of 75% SAC-only treatment) are sufficient for achieving the EPHP goal for S. mansoni.” As indicated in my previous comments, the current guidelines indicate that to achieve elimination of schistosomiasis as a public health problem, WHO recommends to “adjust preventive chemotherapy and to use additional complementary public-health interventions” (World Health Organization, 2013 ^3^). Hence, it remains unclear, why the authors stick to recommendations for morbidity control and not elimination as a public health problem?

See response to comment 2 in abstract and *.

8. The authors write: “As the burden of infection in adults relative to SAC increases…”. The authors might define burden. Is it measured in intensity, worm numbers, DALYs or something else?

*The burden relates to the transmission intensity - The age-specific contact rates, i.e. transmission intensities by age group, were varied such that the adults have a low or high burden of infection relative to SAC. This has been clarified in the sentence referred to: “As the burden of infection (intensity of transmission) in adults relative to SAC increases…”*

**Practical implications:**

1. The authors write: “EPHP is technically feasible in all settings within 10 years provided that the appropriate treatment strategy is used...”. A recent study on S. haematobium in Zanzibar, a setting targeted for elimination as public health problem and interruption of transmission, showed that despite 5 years of bi-annual MDA in schoolchildren with high coverage and adults with moderate compliance, elimination as public health problem was not reached in all sentinel sites, probably due to heterogeneity in transmission and some remaining pockets with high transmission potential, where people got reinfected rapidly (Knopp et al., 2019a ^1^, Knopp et al., 2019b ^2^). The authors might consider validating their model based on available field data.

Agree, we are planning to do this. Our modelling insights here are assuming 100% adherence and coverage at random at each round of MDA (see response ** above).

2. The authors write: “To monitor and assess progress towards the EPHP goal, prevalence and infection intensity data are required from SAC (as the goal is defined by <1% prevalence of heavy-intensity infections in SAC).” The authors might consider adding a reference. When checking the proposed goals published by WHO at https://www.who.int/neglected_diseases/news/NTD-Roadmap-targets-2021-2030.pdf?ua=1, I did not see any mention of “SAC“. The current guidelines define elimination as public health problem as “<1% heavy infection intensities in all sentinel sites” (World Health Organization, 2013 ^3^).

*** We are referring to the current EPHP goal here as stated at the end of the background section. Defined in table 4.4 in reference 4a (reference has been added to letter) as heavy-intensity infections are measured in SAC.

3. The authors write: “The goal is currently assessed by averaging the prevalence measured in five schools randomly sampled within a district.” Is measuring prevalence in 5 schools related to the modelling approach of the authors or is it a recommendation by WHO to? If the latter, the authors might consider providing a reference.

*5 schools per district are frequently surveyed for mapping schistosomiasis. This has been edited to: “The goal is currently typically assessed by averaging the prevalence measured in five schools randomly sampled within a district ^10^.”*

4. The authors write: “Pre-SAC can also be infected with schistosomiasis…”. The authors might consider that a patient is not infected with a disease but with the disease-causing agent, in this case schistosomes.

*Corrected to schistosomes.*

5. The authors write: “Development of a paediatric formulation of praziquantel for pre-SAC treatment would prevent this”. The authors might consider that not only the development but finally only the large-scale application of the paediatric praziquantel would reduce a potential reservoir in PSAC.

*We have not used our modelling analyses to determine the coverage at which paediatric praziquantel would need to be given in order to have an impact. Hence, we do not mention the scale of this.*

6. The authors write: “Due to remaining infections, it is highly likely that treatment will still be needed after achieving EPHP”. Again, it would be nice if the authors can clarify what is meant with “treatment”.

*See response to abstract comment 9.*

7. The authors write: “To alleviate the need for ongoing treatment and to prevent resurgence, EOT is required after reaching EPHP.” The authors might consider indicating that this is/was one of the goals of WHO for selected countries until 2025 and that also “GSA strongly supports the proposal of the ambitious goal of interruption of transmission in selected countries”.

*In the background we state that IOT is the WHO end goal for schistosomiasis. Also see response ^.*

8. The authors write: “The transition of treatment programmes from EPHP to EOT will require reassessment of the treatment strategy.” The authors might consider clarifying if they indeed refer to treatment strategies only, or broader intervention strategies, i.e. including also snail control, WASH, behaviour change, surveillance-response, etc.

*We are referring to MDA and broader intervention strategies. The following has been added to the sentence to clarify: “with consideration of additional interventions such as behaviour change and snail control.”*

**Table 1:**

1. The authors write: “Elimination as a public health problem (EPHP): <1% prevalence of heavy-intensity infections in SAC”. Note that this is the current 2025 goal. To my knowledge, the current goal is not referring to SAC but sentinel sites?

See response ***.

2. The authors write: “In low to moderate prevalence settings (<50% SAC prevalence prior to treatment), EPHP is likely to be achieved with 75% SAC-only treatment.” For how many years is SAC needed to be treated?

*Timelines to achieve EPHP are shown in Table 2.*

**Priority questions:**

- Validation of the models with real data from different prevalence and intensity settings could be one additional aim.

*We agree and this is part of our ongoing work. However, the priority questions listed here are those which have been identified in discussions with WHO.*

# Peer review: Reviewer response for version 1

**DOI:** 10.21956/gatesopenres.14179.r27699

**Published:** 2019-09-04

**Authors:** W. Evan Secor

**Affiliations:** 1 Parasitic Diseases Branch, Division of Parasitic Diseases and Malaria, Centers for Disease Control and Prevention, Atlanta, GA, USA

The Schistosomiasis Group of the NTD Modelling Consortium reports the projected coverage levels necessary to achieve the WHO-defined elimination of schistosomiasis as a public health problem (EPHP, <1% prevalence of heavy-intensity infections). The modelling results demonstrate that this definition of EPHP may be possible (at least under a 10 year program) if sufficient levels of MDA coverage are attained. The challenge comes from how reliable this definition of EPHP is in terms of true elimination of schistosomiasis-associated morbidity. It has never been clearly demonstrated that morbidity is eliminated when there are fewer than 1% heavy infections. This is not the responsibility or intent of the authors but it would be valuable for them to mention the limitations of EPHP as currently defined and that the model requirements for length and minimum coverage of MDA may well need revising once an evidence-based definition of schistosomiasis morbidity elimination has been established.

I confirm that I have read this submission and believe that I have an appropriate level of expertise to confirm that it is of an acceptable scientific standard.

# Response

**Published:** 2019-11-15

**Authors:** Jaspreet Toor

We thank Evan for his feedback and agree with the concerns raised regarding the current WHO definition of EPHP. We have added more about how this current definition is limited in terms of its relationship to the reduction of schistosomiasis-associated morbidity. Further modelling will be done following revision of the goal as this may impact our recommended treatment strategies.

 The following has been added in the background section: “There is uncertainty around how reliable the current WHO definition of EPHP is for estimating a reduction in schistosomiasis-related morbidity as lower intensity infections may also be associated with significant morbidity ^3^. Further modelling will be required following revision of this goal by WHO as this may impact our recommended treatment strategies.”
